# DDAH1 mediates gastric cancer cell invasion and metastasis *via* Wnt/β‐catenin signaling pathway

**DOI:** 10.1002/1878-0261.12089

**Published:** 2017-06-22

**Authors:** Jianxin Ye, Jie Xu, Yun Li, Qiang Huang, Jinsheng Huang, Jinzhou Wang, Wenjing Zhong, Xinjian Lin, Wannan Chen, Xu Lin

**Affiliations:** ^1^ Key Laboratory of Ministry of Education for Gastrointestinal Cancer Fujian Medical University Fuzhou China; ^2^ Department of Gastrointestinal Surgery The First Affiliated Hospital of Fujian Medical University Fuzhou China; ^3^ Fujian Key Laboratory of Tumor Microbiology Fujian Medical University Fuzhou China

**Keywords:** dimethylarginine dimethylaminohydrolase 1, epithelial–mesenchymal transition, gastric cancer, tumor suppressor, Wnt/β‐catenin

## Abstract

Gastric cancer (GC) represents the fourth most common malignant neoplasm and the second leading cause of cancer death. Despite therapeutic advances in recent decades, the clinical outcome remains dismal owing to the fact that most patients with GC show advanced disease at diagnosis and current chemotherapy only confers a modest survival advantage. Identification of key molecular signaling pathways involved in gastric carcinogenesis and progression would aid in early diagnosis and provide a rational design for targeted therapies in selected patients with advanced GC, to improve their outcome. Dimethylarginine dimethylaminohydrolase 1 (DDAH1) is the main enzyme that can degrade asymmetric dimethylarginine, an endogenous nitric oxide synthase (NOS) inhibitor. Increased DDAH1 expression and NO production have been linked to multiple pathological conditions including cancer. However, the prognostic significance of DDAH1 in patients with GC and its function in GC progression remain undefined. In this study, we found that downregulation of DDAH1 was frequently detected in GC tissues and strongly correlated with more aggressive phenotypes and poor prognosis. Functional assays confirmed that forced expression of DDAH1 in the GC cells suppressed cell migration and invasion *in vitro*, as well as metastatic potential *in vivo*. DDAH1 overexpression inhibited the epithelial–mesenchymal transition process by increasing β‐catenin degradation through the attenuation of Wnt/GSK‐3β signaling. In contrast, knockdown of DDAH1 produced the opposite effect. These findings suggest that DDAH1 functions as a tumor suppressor in GC and may be exploited as a diagnostic and prognostic biomarker for GC.

**Abbreviations**
DDAH1: dimethylarginine dimethylaminohydrolase 1
EMT: epithelial–mesenchymal transition
GC: gastric cancer

## 1 Introduction

Gastric cancer (GC) is one of the most prevalent cancer types and the second leading cause of oncologic deaths worldwide. About seventy percentage of these cases occur in developing countries, especially in East Asia (Ferlay *et al*., 2015; Hartgrink *et al*., 2009; Parkin *et al*., 2005). Recently, it has become possible to cure GC if it can be detected early. However, the majority of patients with GC are usually diagnosed at an advanced stage leading to poor clinical outcomes (Hohenberger and Gretschel, 2003). Despite the rapid therapeutic advances, the 5‐year survival rate of GC is still low (Karimi *et al*., 2014; Sehdev and Catenacci, 2013). Therefore, identification and better understanding of novel biomolecules and signaling pathways involved in the gastric carcinogenesis and progression remain of great importance.

Dimethylarginine dimethylaminohydrolase 1 (DDAH1) is the main enzyme that metabolizes asymmetric dimethylarginine, an endogenous inhibitor of nitric oxide synthase (NOS; Kostourou *et al*., 2002). Consequently, DDAH1 could indirectly increase NO. Numerous studies have demonstrated that overexpression of DDAH1 promotes angiogenesis *in vitro* and increases glioma growth *in vivo* through enhanced expression of NO and VEGF (Kostourou *et al*., 2002; Smith *et al*., 2003; Wojciak‐Stothard *et al*., 2007). DDAH1 also plays a vital role in early embryogenesis (Breckenridge *et al*., 2010; Leiper *et al*., 2007). More recently, a study has shown that DDAH1 can inhibit the renal fibrosis process *via* the Wnt/β‐catenin pathway (Liu *et al*., 2016), a well‐established crucial signaling event associated with a number of human diseases (Miao *et al*., 2013; Miki *et al*., 2011; de Sousa *et al*., 2011) including cancer (Ramachandran *et al*., 2012). Wnt signaling regulates various functions of the cancer cell such as proliferation, cell fate decision, motility and invasion through activating β‐catenin (Clevers, 2006). A large body of evidences have suggested that the canonical Wnt/β‐catenin pathway plays an important role in inducing epithelial cancer cells to undergo epithelial–mesenchymal transition (EMT; Brabletz *et al*., 2005; Taki *et al*., 2003), which facilitates migration through the extracellular matrix and distant metastasis.

However, the role of DDAH1 in GC development and progression is unclear, and the mechanisms underlying DDAH regulation of malignant transformation remain to be elucidated. In this study, we evaluated the clinical significance of the expression of DDAH1 in a cohort of 150 human GC specimens and analyzed its correlation with clinicopathological features and survival of the patients. We further investigated the functional impact and underlying molecular mechanisms of DDAH1 on GC progression in a series of *in vitro* and *in vivo* assays.

## 2 Materials and methods

### 2.1 Cell lines and clinical samples

Seven human GC cell lines (NCI‐N87, MKN74, AGS, NUGC‐3, MKN45, MGC803, and HGC‐27) were obtained from the Type Culture Collection of the Chinese Academy of Sciences (Shanghai, China). All cell lines were maintained in RPMI‐1640 (Gibco BRL, Grand Island, NY, USA) supplemented with 10% fetal bovine serum (FBS) except AGS in Ham's F12 medium (Cellgro, Manassas, VA, USA) and incubated at an atmosphere containing 5% CO_2_ at 37 °C. Human GC samples and their corresponding nontumorous gastric tissues were collected at the time of surgical resection at The First Affiliated Hospital of Fujian Medical University (Fuzhou, China) from 2008 to 2010. The tissues were immediately frozen in liquid nitrogen and stored at −80 °C freezer or fixed in 10% formalin for paraffin embedding. All samples were collected with patients’ informed consent, and the study was approved by the institutional review board and regulatory authorities of Fujian Medical University. Clinicopathological classification and staging were determined according to American Joint Committee on Cancer seventh edition of GC TNM staging (Wittekind, 2010). No patients had received chemotherapy or radiotherapy before surgery.

### 2.2 Tissue microarray and immunohistochemistry

A tissue microarray was constructed using two cores of 1 mm in diameter per sample from the 150 patients with GC. Immunohistochemistry (IHC) studies were performed on formalin‐fixed, paraffin‐embedded tissue microarrays using human anti‐DDAH1 antibody (1 : 200; Abcam, Cambridge, UK) and β‐catenin antibody (1 : 100; Cell Signaling, Danvers, MA, USA). The degree of DDAH1 staining was quantified according to the following calculation: the score of stained tumor cells (0, ≤ 5% positive cells; 1, 5–25% positive cells; 2, 26–50% positive cells; 3, 51–75% positive cells; 4, ≥ 75% positive cells) multiplied with the score of staining intensity (0, no staining; 1, weak staining, light yellow; 2, moderate staining, yellow brown; 3, strong staining, brown) to obtain a final score ranging from 0 to 12. A final score of 3 or less was classified as low‐expression group, while 4–12 as high‐expression group. β‐Catenin staining was considered positive if > 10% of the tumor cells showed yellow or brown staining.

### 2.3 Western blot analysis

Tissues or cells were lysed in Western and IP cell lysis buffer (Beyotime, Shanghai, China) with PMSF (Amresco, Solon, Ohio, USA) for 30 min on ice at 4 °C, followed by centrifugation at 12 000 ***g*** for 10 min at 4 °C. The supernatants were collected as total proteins and then measured using the BCA Protein Assay Kit (Thermo Scientific, Waltham, MA, USA). The same amount of proteins in each well were separated with 10% SDS/PAGE and transferred to a 0.45‐μm PVDF membrane (Amersham Hybond; GE Healthcare, München, Germany). Then, the membrane was blocked in 0.5% albumin from bovine serum (Amresco) followed by incubation overnight at 4 °C with the primary antibodies against DDAH1 (1 : 2000; Abcam), E‐cadherin (1 : 1000; Cell Signaling), ZO‐1 (1 : 1000; Cell Signaling), vimentin (1 : 1000; Cell Signaling), N‐cadherin (1 : 1000; Cell Signaling), Snail (1 : 1000; Cell Signaling), β‐catenin (1 : 1000; Cell Signaling), GSK‐3β (1 : 2000; Cell Signaling), p‐GSK‐3β (Ser9; 1 : 1000; Cell Signaling), p‐β‐catenin (Ser33/37/Thr41; 1 : 1000; Cell Signaling), laminB (1 : 2000; Cell Signaling), Wnt1 (1 : 200; Santa Cruz Biotechnology, Santa Cruz, CA, USA), or β‐actin (1 : 2000; Sigma‐Aldrich, St. Louis, MO, USA). After three washes for 10 min each in TBST, the membrane was further incubated with the secondary antibodies for 1 h at room temperature, and the blots were developed using enhanced chemiluminescence (Lulong Biotech, Xiamen, China).

### 2.4 RNA extraction and real‐time quantitative PCR

Total RNA was isolated from cell lines or frozen tissues with Qiagen RNeasy kit according to the manufacturer's instruction. 1 mg RNA was reverse‐transcribed using miScript Reverse Transcription Kit (Qiagen, Hilden, Germany) for the first‐strand complementary DNA synthesis. Quantitative PCR was performed using SYBR Premix EX Taq kit (Takara, Shiga, Japan). The specific primers were used to detect the relative mRNA expression of DDAH1, β‐catenin, E‐cadherin, ZO‐1, vimentin, N‐cadherin, and Snail by the $2^{-\Delta \Delta C_{t}}$ method. The expression level was normalized against endogenous β‐actin. All the primers were designed by BioSune Biotechnology Co., Ltd (Shanghai, China; Table S1).

### 2.5 Plasmids and establishment of stably transduced GC cell lines

The open reading frame of human DDAH1 (GenBankNM_012137.3) was constructed into the eukaryotic expression vector pCDH (Invitrogen, Carlsbad, CA, USA). The recombinant plasmid pCDH‐DDAH1, lentivirus plasmids pMDL, p‐VSV‐G, and p‐REV (Invitrogen) were cotransfected in 293T at 30% confluence in 10‐cm culture dishes. The medium was collected after filtration with 0.45‐μm filters and added into the culture of GC cells (AGS and MGC803) in 6‐cm culture dishes. After incubation for 48 h, puromycin at 2 μg·mL^−1^ (Sigma‐Aldrich) was used to select stably transduced cells for 7–14 days. Stable DDAH1‐overexpressing clones (AGS‐pDDAH1 or MGC803‐pDDAH1) and the empty vector‐transfected cells (AGS‐pCDH or MGC803‐pCDH) were used for further study. For the generation of DDAH1‐knockdown clones, two shRNA sequences targeting different sites of DDAH1 were synthesized and cloned into pSuper‐retro‐puro plasmid (Oligoengine, Seattle, WA, USA). The recombinant plasmid or empty vector was cotransfected with packaging plasmids pIK (Invitrogen) into 293T cells. The supernatants were collected and used to infect AGS or MGC803 cells. The puromycin‐resistant clones were expanded into cell lines as DDAH1‐knockdown cells (AGS‐psh1DDAH1, AGS‐psh2DDAH1, MGC803‐psh1DDAH1, MGC803‐psh2DDAH1) or the control cells (AGS‐pSuper or MGC803‐pSuper). To rescue DDAH1 expression in the DDAH1‐knockdown AGS and MGC803 cells, six synonymous point mutations were introduced into the shRNA target regions in the full‐length DDAH1 cDNA. The shRNA‐targeted sequence in DDAH1 was CGC TAC GAC AAA CTC ACT; the mutations introduced in the shRNA‐targeted sequence of DDAH1 were CGA TAT GAT AAG CTA ACC. The shRNA‐resistant cDNA was subcloned into pcDNA3.1 vector for transfection. The oligonucleotides and primers used are listed in Table S1.

### 2.6 Cell proliferation assay

Cell proliferation was assessed using the Cell Counting Kit‐8 (CCK‐8; Dojindo, Kuma‐moto, Japan). Cells were seeded at a density of 1000 cells per well in 96‐well plates and incubated at 37 °C, 5% CO_2_ for 24, 48, 72, 96 or 120 h. 10 μL of CCK‐8 solution was added into each well and incubated at 37 °C for 4 h. The absorbance at 450 nm was measured using a microplate reader (Bio‐Tek, Winooski, VT, USA).

### 2.7 Colony formation assay

5 × 10^2^ cells were plated in 60‐mm plates with Ham's F12 or RPMI‐1640 containing 10% FBS and cultured for 14 days. Colonies were stained with crystal violet for 5 min, and the number of colonies containing 50 or more cells was counted. For soft agar colony formation assay, 2 ×Ham's F12 or RPMI‐1640 containing 20% FBS and 2 × 10^3^ cells was mixed with equal volume of 0.7% agarose and immediately plated in six‐well plates containing an underlayer of 0.5% agarose made in 1×Ham's F12 or RPMI‐1640 supplemented with 10% FBS. The plates were cultured at 37 °C under 5% CO_2_ for 14–21 days. Surviving colonies (> 50 cells per colony) were counted and photographed with a Qimaging Micropublisher 5.0 RTV microscope camera (Olympus, Tokyo, Japan).

### 2.8 Cell migration and invasion assay

For the migration assay, 6 × 10^4^ cells in serum‐free media were plated into the upper chamber of Transwell insert (8‐mm pore size; BD Biosciences, San Jose, CA, USA). For the invasion assay, the Transwell insert was coated with Matrigel (BD Biosciences) and 1 × 10^5^ cells were plated onto the top of the coated insert. The medium containing 20% FBS in the lower chamber served as chemoattractant. After 24 h of incubation at 37 °C in a 5% CO_2_ humidified incubator, the cells that did not migrate or invade through the pores in the upper chambers were removed with a cotton swabs and then the lower surface of the filter was stained with 0.1% crystal violet in 20% methanol for 10 min, imaged, and counted using a Qimaging Micropublisher 5.0 RTV microscope camera (Olympus).

### 2.9 Wound‐healing assay

Cells were plated into six‐well plates and grown to nearly 100% confluence. A same size scratch was made through the cell monolayer using a 200‐μL disposable pipette tip. After washing with PBS, fresh culture medium was added and the cells were incubated at 37 °C in 5% CO_2_. Wound closure was photographed at 0, 36 or 48 h.

### 2.10 Animal studies

All work performed with animals was approved by the Institutional Animal Care and Use Committee at the Fujian Medical University. For *in vivo* tumor growth study, five BALB/c nude mice at the age of 4–6 weeks were subcutaneously inoculated with 2 × 10^6^ AGS‐pDDAH1 and AGS‐pCDH cells bilaterally into the left and right flank region. After the tumors were palpable, the tumor volume was determined every 4 days by the formula: length × width^2^/2, and then plotted as a function of time to generate the *in vivo* growth curves. On day 24 after implantation, all animals were euthanized and the tumors were removed, photographed, and weighed.

For the *in vivo* metastasis study, 2 × 10^6^ AGS‐pDDAH1 or AGS‐pCDH cells were resuspended in 0.1 mL serum‐free Ham's F12 and injected into the tail veins of ten BALB/c nude mice. At 8 weeks postinjection, 5 mice in each group were euthanized and the number of tumor nodules formed on the lung and liver surfaces was counted. Lungs and livers were excised and embedded in paraffin for hematoxylin and eosin staining. The remaining five mice were continuously observed until death was recorded for all the five animals.

### 2.11 Dual luciferase reporter assay

The TCF‐responsive luciferase constructs, Top‐Flash and its mutant Fop‐Flash (Addgene, Cambridge, CA, USA), were used to study β‐catenin transcriptional activity. Twelve hours after GC cells were seeded into 24‐well plates, the cells were then cotransfected with Top‐Flash and pRL‐TK (Addgene) report vector or Fop‐Flash and pRL‐TK report vector. The relative luciferase activity was determined using a dual luciferase reporter assay kit (Promega, Madison, WI, USA). The PRL‐TK report vector was used as an internal control.

### 2.12 Immunofluorescent staining

Cells were plated on 8‐μm‐thick chip, fixed in 4% ice‐cold paraformaldehyde, and permeabilized using 0.5% Triton X‐100/PBS for 20 min at room temperature. The cells were blocked with 10% albumin from bovine serum (Amresco) for 1 h and then incubated with antibodies against vimentin, E‐cadherin (1 : 50; Cell Signaling), DDAH1, and β‐catenin (1 : 50; Abcam) overnight at 4 °C. After three washes, cells were incubated with Alexa Fluor 488‐conjugated goat anti‐rabbit secondary antibody (1 : 200; Invitrogen) or Alexa Fluor 647‐conjugated goat anti‐mouse IgG (1 : 200; Abcam). The nuclei were counterstained with DAPI (Invitrogen), and cells were visualized with a laser scanning confocal microscope (Leica Microsystems, Wetzlar, Germany).

### 2.13 Statistical analysis

Statistical analysis was performed using spss 22.0 for Windows. Student's *t*‐test was used to determine the statistical differences in each two‐group comparison. All data were presented as means ± SD from three independent assays. Associations between DDAH1 expression and the clinicopathological parameters of the patients with GC were analyzed using the Pearson χ^2^ test. The survival curves were plotted using Kaplan–Meier analysis. Differences were considered significant when *P *< 0.05.

## 3 Results

### 3.1 DDAH1 is downregulated in human GC and its low expression correlates with the patients’ poor clinical outcome

**Figure 1:**
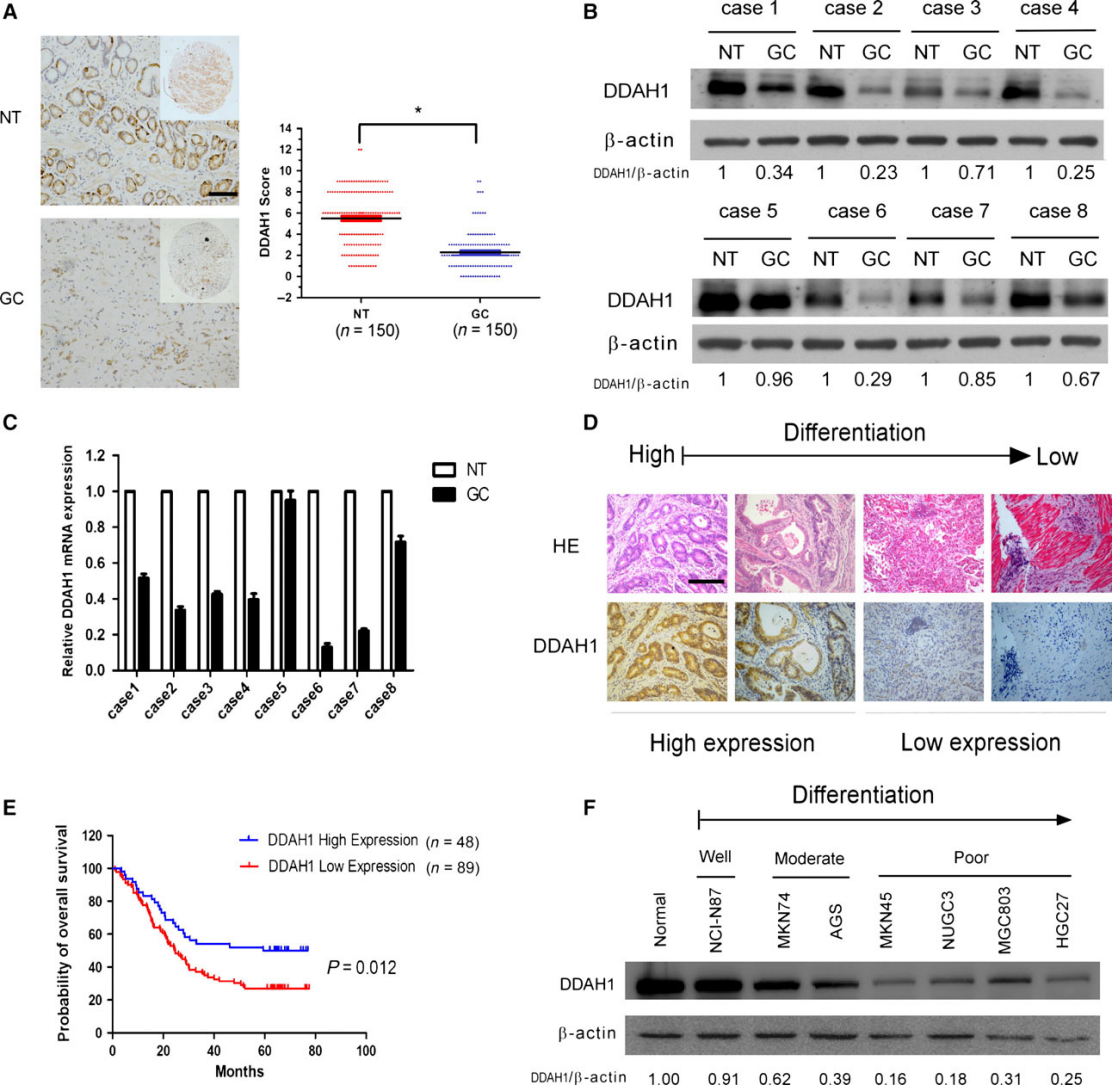
DDAH1 was downregulated in gastric carcinoma and inversely correlated with prognosis. (A) Expression of DDAH1 in GC tissues of 150 patients and their corresponding noncancerous mucosal tissues (NT) was analyzed by IHC. Scale bar, 50 μm. The graph shows the scores of DDAH1 in GC and NT ranging from 0 to 12. Paired *t*‐test was used to compute *P*‐value (*P < 0.05). (B, C) Western blot analysis and real‐time qPCR results showing the protein and mRNA level of DDAH1 in GC tissues and the corresponding noncancerous mucosal tissues from randomly chosen eight patients. β‐Actin served as a loading control. (D) Hematoxylin and eosin staining showing different differentiation of GC tumors and corresponding DDAH1 expression. Scale bar, 50 μm. (E) Kaplan–Meier survival analysis of 137 GC patients with high or low DDAH1 (*P *= 0.012, log‐rank test). (F) DDAH1 protein expression in seven GC cell lines and normal gastric mucosa (NGM). β‐Actin served as a loading control.

First, we investigated DDAH1 expression in 150 human GC tissues and the corresponding adjacent normal tissues (NT) using IHC. As shown in Fig. 1A and Table 1, expression of DDAH1 was significantly lower in GC tumors compared with their corresponding adjacent normal tissue. Among 150 pairs of samples, 67.3% of GC tissues showed low DDAH1 expression, whereas only 18.7% of the corresponding adjacent normal tissue expressed the low level of DDAH1. Western blot analysis and qRT‐PCR also confirmed a lower expression of both DDAH1 protein (Fig. 1B) and mRNA level (Fig. 1C) in the gastric cancer tissues as compared to the adjacent normal tissues. Correlation analysis of DDAH1 expression with patients’ clinicopathological characteristics (Table 2) demonstrated that DDAH1 expression was negatively associated with the presence of lymph node metastasis (*P* = 0.034) and diffuse classification (*P* = 0.013), but positively with differentiation status (*P* = 0.003, Fig. 1D). The rest of pathologic variables including gender, age, tumor size, depth of tumor invasion, clinical stage, lymphovascular invasion and nerve invasion exhibited no significant association with DDAH1 expression. Kaplan–Meier survival analysis showed that patients with low DDAH1‐expressing tumors had significantly shorter survival than patients with high DDAH1‐expressing tumors (Fig. 1E, median survival time 24.6 months versus 59.4 months). To further ascertain the downregulation of DDAH1 in GC, expression of DDAH1 in seven GC cell lines was examined by western blot analysis. Consistent with the results obtained from primary GC tissues, DDAH1 was downregulated in all the GC cell lines examined (Fig. 1F). Notably, DDAH1 expression level was also positively correlated with the differentiation status of the cells. Taken together, these results indicate that DDAH1 may function as a tumor suppressor and downregulation of it may promote the development and progression of GC.

**Table 1:** DDAH1 expression in 150 pairs of GC samples and their corresponding adjacent normal tissue

Tissues	DDAH1		*P*‐value
	Low expression	High expression	
GC (*n *= 150)	101	49	< 0.001
NT (*n *= 150)	28	122	

**Table 2:** Clinicopathological characteristics of 150 patients with GC according to DDAH1 expression

Characteristics	Total	DDAH1 expression		*P*‐value
		Low	High	
Age (years)				
< 60	58	43	15	0.158
≥ 60	92	58	34	
Gender				
Male	110	70	40	0.109
Female	40	31	9	
Tumor size (cm)				
< 5	79	58	20	0.056
≥ 5	71	43	29	
Depth of tumor invasion				
T2	11	8	3	0.910
T3	65	43	22	
T4	74	50	24	
Lymph node metastasis				
N0	24	13	11	**0.034**
N1	31	17	14	
N2	31	20	11	
N3	64	51	13	
Clinical stage				
I/II	49	28	21	0.064
III/IV	101	73	28	
Differentiation				
High/middle	57	30	27	**0.003**
Low/undifferentiation	93	71	22	
Lauren classification				
Intestinal	47	25	22	**0.013**
Diffuse	103	76	27	
Lymphovascular invasion				
Absent	78	53	25	0.867
Present	72	48	24	
Nerve invasion				
Absent	98	65	33	0.781
Present	52	36	16	

Bold values indicate statistical significance with *P* < 0.05.

### 3.2 Effect of DDAH1 on GC cell proliferation, migration, invasion, and metastatic potential

**Figure 2:**
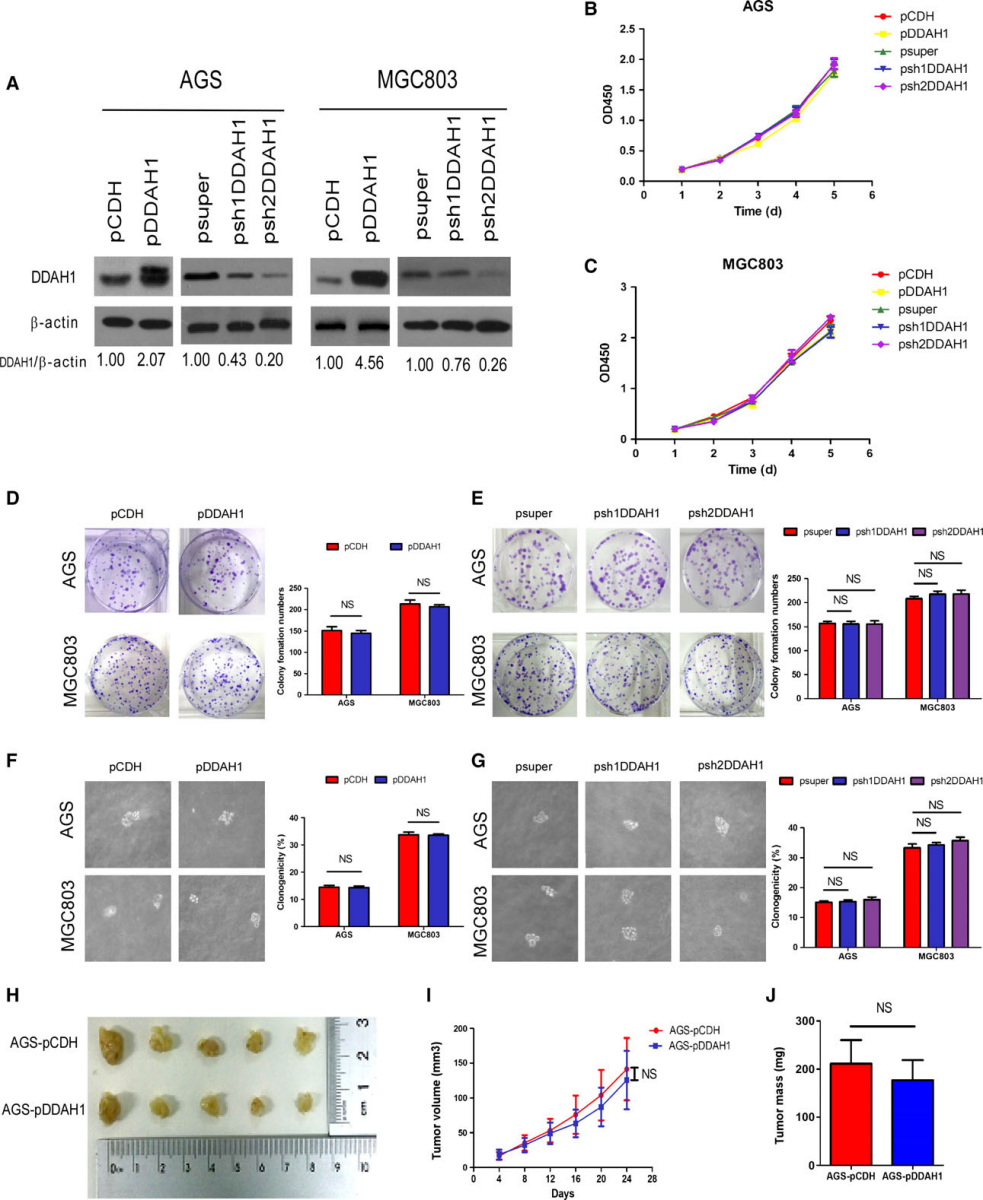
DDAH1 did not affect GC cell growth *in vitro* and *in vivo*. (A) Western blot analysis confirming stable overexpression or knockdown of DDAH1 in AGS and MGC803. β‐Actin was used as a loading control. (B, C) CCK8 assay showing no effect of DDAH1 on the proliferation rate of AGS or MGC803 cells. Data were expressed as mean ± SD of three independent experiments (NS indicates no statistical significance). (D, E) Colony formation assay showing no effect of DDAH1 on the colony‐forming ability of AGS or MGC803 cells. Data were expressed as mean ± SD of three independent experiments (NS indicates no statistical significance). (F, G) Soft agar colony formation assay showing no effect of DDAH1 on the clonogenicity of AGS or MGC803 cells. Data were expressed as mean ± SD of three independent experiments (NS indicates no statistical significance). (H) Photograph of the xenografts removed from nude mice at 24 days after implantation. (I) *In vivo* tumor growth curves showing that DDAH1 did not influence xenograft tumor growth in nude mice. Points and bars represent mean ± SD from five mice per group (NS indicates no statistical significance). (J) Tumor weight of two groups was measured. Data were expressed as mean ± SD (NS indicates no statistical significance).

Given the evidence that DDAH1 expression was of prognostic relevance in GC, next we sought to determine the functional role of DDAH1 in GC malignant behaviors both *in vitro* and *in vivo*. We used both gene silencing and overexpression strategies to specifically knock down or overexpress DDAH1 in the GC cell lines AGS and MGC803. Stable overexpression or knockdown of DDAH1 in these cells was confirmed by western blot analysis (Fig. 2A). Overexpression or silencing of DDAH1 did not affect the proliferation rate of AGS or MGC803 cells as evaluated by CCK‐8 assay (Fig. 2B,C), either by colony formation assay (Fig. 2D,E) or by soft agar colony formation assay (Fig. 2F,G). Similar to the results obtained from the *in vitro* growth assays, DDAH1 did not affect the growth of GC xenografts *in vivo* (Fig. 2H–J).

**Figure 3:**
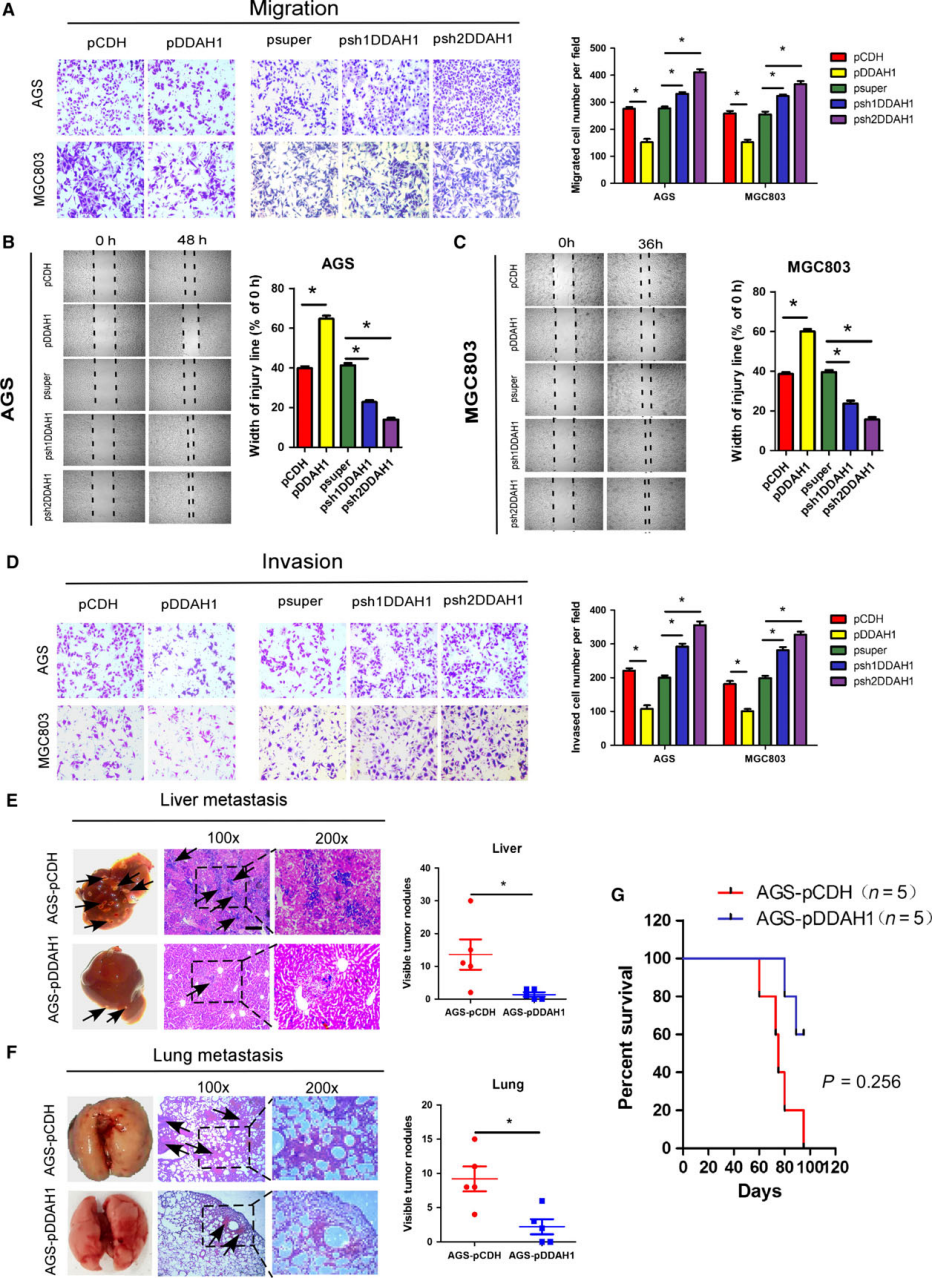
DDAH1 regulated GC cell migration, invasion, and metastatic potential. (A) Relative migration of the GC cells through an uncoated filter toward serum‐containing medium in a Boyden chamber assay. (B, C) Relative motility as determined by the ability of the GC cells to close a wound made by creating a scratch through a lawn of confluent cells. (D) Relative invasion of the GC cells through a layer of Matrigel coated on the filter of a Boyden chamber. (E, F) Liver and lung metastatic burden in mice 8 weeks after tail vein injection of the GC cells. Scale bar, 50 μm. *P < 0.05. (G) Kaplan–Meier survival analysis showing that DDAH1‐overexpressing group had longer survival time than the negative control group (*P* = 0.0256).

The effect of DDAH1 on cell migration was compared using a Boyden two‐chamber assay in which the cells were induced to migrate through a filter by the presence of FBS on the opposite side. As shown in Fig. 3A, overexpression of DDAH1 suppressed the cell migratory ability, whereas DDAH1 knockdown enhanced the migration. A wound‐healing/scratch assay was also used to confirm the changes in cell migration as the extent of wound closure can be taken as a direct measure of cell motility. As shown in Fig. 3B,C, upregulation of DDAH1 remarkably attenuated the wound closure, whereas DDAH1‐knockdown cells closed the wound more rapidly than the control cells. The invasive potential of the GC cells was assessed by a modified Boyden chamber invasion assay. Likewise, overexpression of DDAH1 significantly decreased the number of cells that invaded through Matrigel compared with the empty vector‐transfected pCDH cells. In contrast, knockdown of DDAH1 in AGS and MGC803 cells led to a significant increase in their invasive ability (Fig. 3D). From the functional studies, we identified that sh2DDAH1 had greater effects than sh1DDAH1 on producing the phenotypic changes; therefore, it was chosen for subsequent experiments.

To determine whether changes in the cell migration and invasiveness detected *in vitro* could translate into differences in metastatic potential *in vivo*, 2 × 10^6^ AGS‐pDDAH1 or AGS‐pCDH cells were injected into the tail vein of BALB/c nude mice and evidence of metastasis to the liver and lungs was examined 8 weeks after the injection. As shown in Fig. 3E, the number of nodules formed in the liver of the mice injected with DDAH1‐overexpressing AGS‐pDDAH1 cells was remarkably less compared to the mice injected with the control AGS‐pCDH cells. Although no visible metastatic nodules were observed on the surface of the lungs, the micrometastases in the lungs can be detected by hematoxylin and eosin staining that showed a significant reduction in the lung metastases in the mice injected with AGS‐pDDAH1 cells compared with the mice injected with the control AGS‐pCDH cells (Fig. 3F). Consistent with the *in vivo* metastasis data, all the five mice injected with AGS‐pCDH cells died within 95 days, whereas 60% of the mice injected with AGS‐pDDAH1 survived for that duration (Fig. 3G). Collectively, these results provide compelling evidence for an important role of DDAH1 in GC progression.

### 3.3 DDAH1 suppresses epithelial–mesenchymal transition

**Figure 4:**
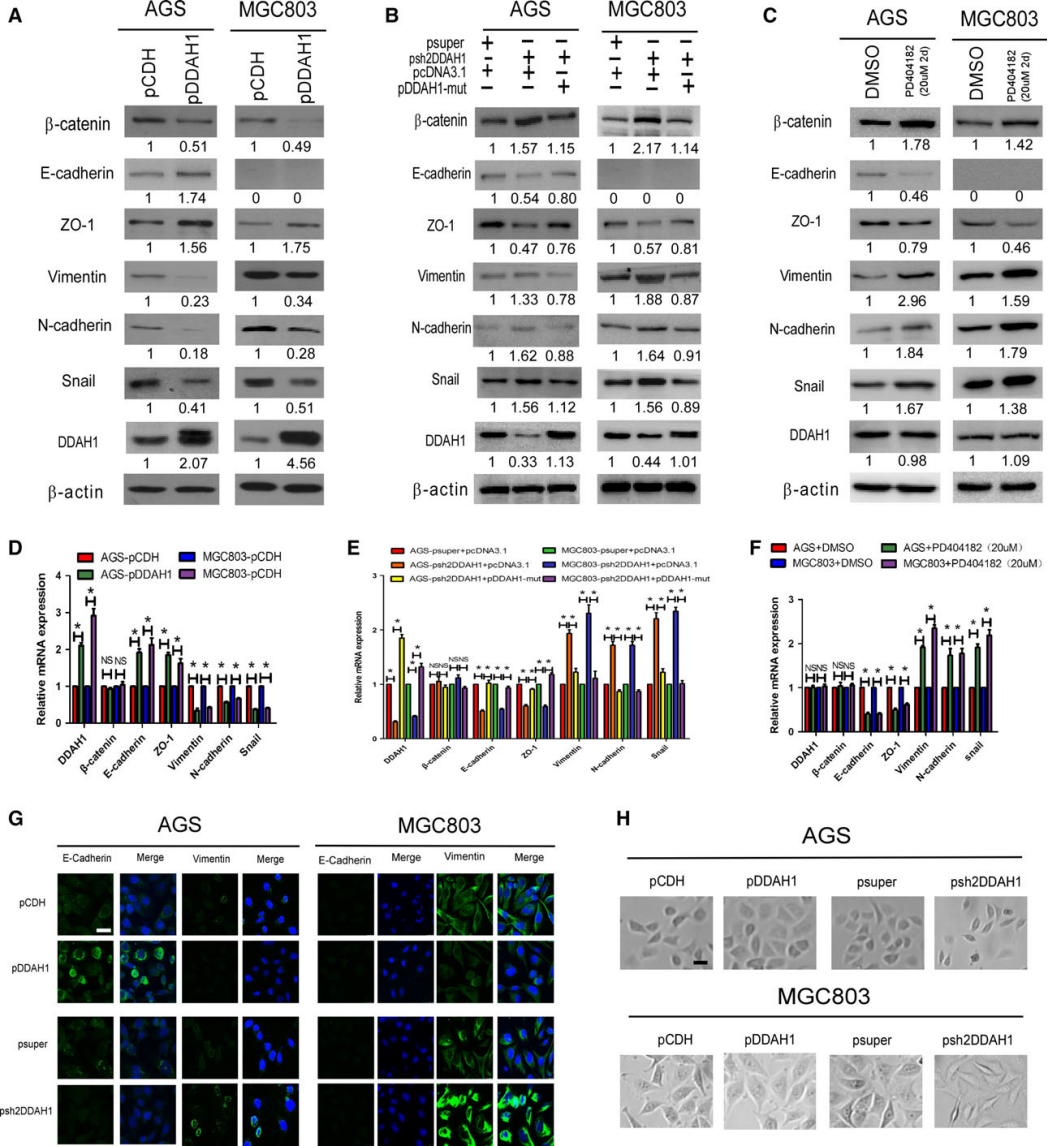
DDAH1 regulated the expression of EMT markers in the GC cells. (A–C) Western blot analysis of the expression of EMT markers in the DDAH1‐overexpressing, shRNAi‐knockdown, or DDAH1‐specific inhibitor PD404182‐treated AGS and MGC803 cells. pDDAH1‐mut represents the vector expressing DDAH1 mRNA that had been mutated to prevent the shRNAi from causing their degradation. (D–F) qRT‐PCR analysis of the expression of EMT markers in the DDAH1‐overexpressing, shRNAi‐knockdown, or DDAH1‐specific inhibitor PD404182‐treated AGS and MGC803 cells. (G) Immunofluorescent staining of E‐cadherin and vimentin expression in the DDAH1‐overexpressing or DDAH1‐knockdown AGS and MGC803 cells. Scale bar, 25 μm. (H) Bright field images of the morphology of DDAH1‐overexpressing or DDAH1‐knockdown cells. Scale bar, 50 μm.

EMT is considered as a crucial event in cancer metastasis. To determine whether the observed phenotypic changes in malignant behaviors of the GC cells as a result of DDAH1 alteration were related to EMT, expression of markers associated with EMT was quantified in the GC cell lines by western blot analysis and qRT‐PCR. As shown in Fig. 4A–F, overexpression of DDAH1 in AGS and MGC803 cells upregulated the expression of epithelial markers (E‐cadherin and ZO‐1) but downregulated the expression of both mesenchymal markers (vimentin and N‐cadherin) and EMT repressor Snail at both protein and mRNA levels. In contrast, knockdown of DDAH1 produced the opposite effect, while E‐cadherin protein level was not detectable in the MGC803 cells. The observation that knockdown of DDAH1 increased both protein and mRNA levels of β‐catenin as well as Snail 1 mRNA level further supports the notion that β‐catenin and Snail are likely to be downstream targets of DDAH1. To confirm the specificity of the effect of DDAH1 knockdown on the expression of β‐catenin and other EMT markers, DDAH1 expression was restored in the AGS‐pshDDAH1 and MGC803‐pshDDAH1 cells by transfecting them with a pcDN3.1 vector expressing DDAH1 mRNA that had been mutated to prevent the shRNA from causing degradation. As expected, re‐expression of DDAH1 in the AGS‐pshDDAH1 and MGC803‐pshDDAH1 cells reversed the expression of β‐catenin and EMT markers to a pattern similar to what was observed in the control cells (Fig. 4B). To extend the findings from DDAH1 knockdown, DDAH1‐specific inhibitor PD404182 was also applied to examine the effect of enzymatic inhibition of DDAH1 on the expression of β‐catenin and EMT markers. As anticipated, pharmacological inhibition of DDAH1 activity produced essentially the same effects as DDAH1 knockdown (Fig. 4C). Furthermore, immunofluorescent study confirmed that DDAH1 was positively correlated with E‐cadherin expression but negatively with vimentin (Fig. 4G). Bright field images of the morphology of DDAH1‐overexpressing or DDAH1‐knockdown cells demonstrated that knockdown of DDAH1 expression induced morphological changes reminiscent of EMT as the cells lost their epithelial cobblestone‐like morphology to acquire a more elongated fibroblast‐like shape. In contrast, overexpression of DDAH1 endowed the cells with more characteristic epithelial morphological features (Fig. 4H). These data strongly implicate that EMT process contributes to GC cell migration and invasion upon the loss of DDAH1 expression.

### 3.4 DDAH1 inversely regulates β‐catenin expression in GC and mediates EMT through β‐catenin

**Figure 5:**
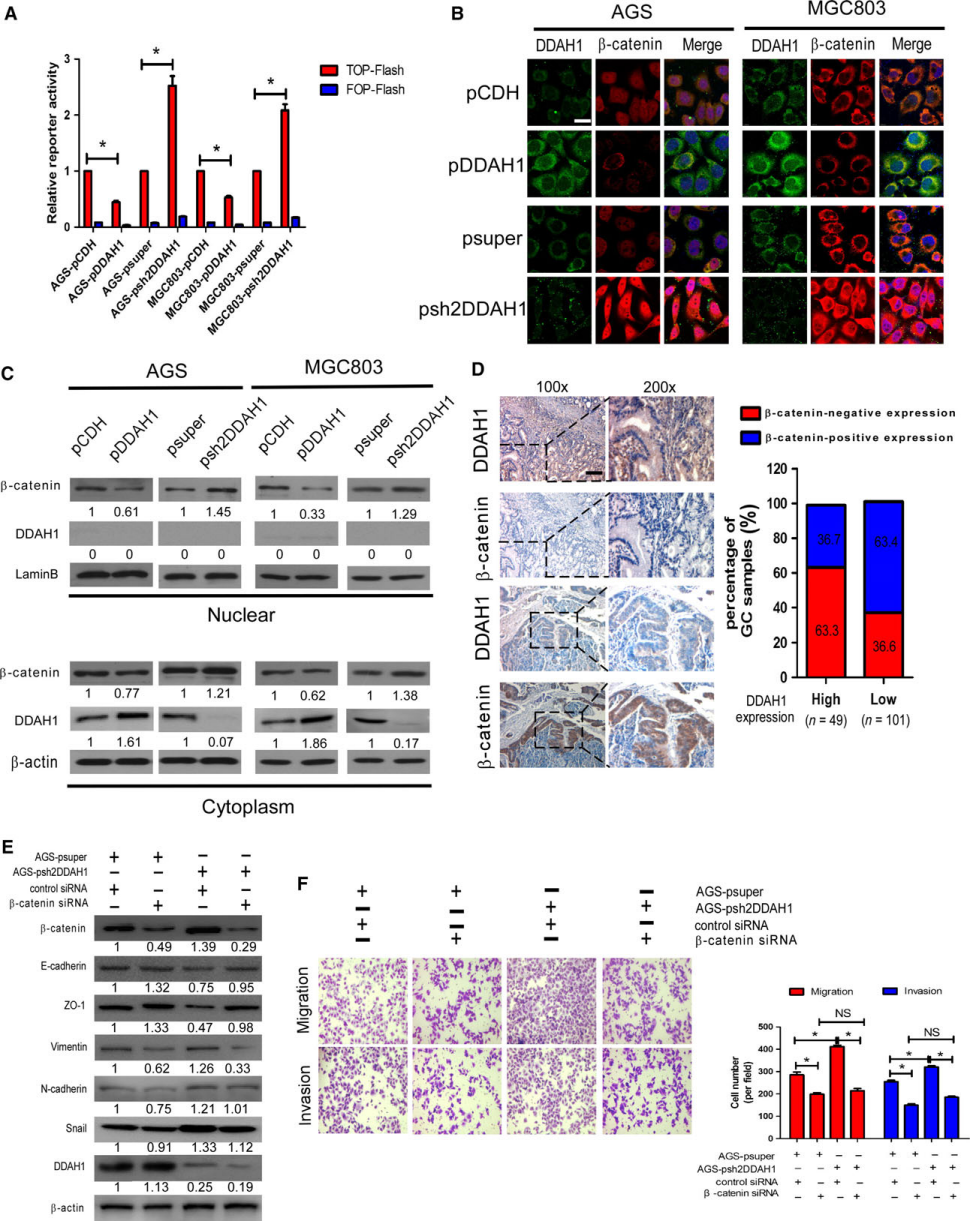
DDAH1 regulated β‐catenin expression in GC and mediated EMT through β‐catenin. (A) DDAH1 expression inversely correlated with β‐catenin activity in TOP‐Flash reporter assay. Expression was normalized with Renilla luciferase activity. The results was performed in three independent experiments (*P < 0.05). (B) Coimmunofluorescence staining showing that nuclear and cytoplasmic β‐catenin was negatively correlated with DDAH1 expression. Expression of DDAH1 was shown in green and β‐catenin in red. DAPI was used as a nuclear counterstain. Scale bar, 25 μm. (C) Fractionation experiments demonstrated that upregulation of DDAH1 decreased the protein levels of nuclear and cytoplasmic β‐catenin in AGS and MGC803, while downexpression of DDAH1 enhanced the nuclear and cytoplasmic β‐catenin expression. Nuclear and cytoplasmic protein loading was normalized for equal levels of laminB and β‐actin. (D) Relationship between DDAH1 expression and β‐catenin cytoplasmic/nuclear accumulation in the 150 GC samples using IHC. Scale bar, 50 μm. In the GC samples with high DDAH1 expression, the percentage of β‐catenin‐positive expression was 36.7%, significantly lower than those with low DDAH1 expression (63.4%). (E) The DDAH1‐silenced stable cells and negative control cells were transfected with β‐catenin siRNA or control siRNA. Western blot analysis showed the effect of β‐catenin on the expression of EMT markers and Snail in the cells. (F) The stimulatory effect of shDDAH1 on the GC cell migration and invasion was blocked by β‐catenin siRNA. The representative images were shown, and the data were expressed as mean ± SD (*P < 0.05; NS, no statistical significance).

Wnt/β‐catenin pathway is well known to play an important role in inducing EMT. To investigate whether DDAH1 affected β‐catenin transcriptional activity in GC, Top‐Flash reporter assays were used to assess the effect of DDAH1 on β‐catenin activity. DDAH1 overexpression significantly reduced the transcriptional activity of β‐catenin, while knockdown of DDAH1 increased the activity (Fig. 5A). Then, we used fluorescent microscopy to detect the intracellular localization of both DDAH1 and β‐catenin. As shown in Fig. 5B, DDAH1 is distributed diffusely in the cytoplasm only, whereas β‐catenin could be found both in the cytoplasmic and in nuclear area. Similarly, overexpression of DDAH1 was accompanied by reduction in nuclear and cytoplasmic β‐catenin, whereas silencing of DDAH1 increased nuclear and cytoplasmic β‐catenin protein level. Fractionation experiment also confirmed a decrease in β‐catenin in the cytoplasm and nuclei of DDAH1‐overexpressing cells but an increase in nuclear and cytoplasmic β‐catenin in the DDAH1‐knockdown cells (Fig. 5C). To further confirm the negative regulation of DDAH1 on β‐catenin expression, immunohistochemical staining was performed on the GC tissues. As shown in Fig. 5D, DDAH1 expression was reversely correlated with the expression of β‐catenin. Given the firm evidence that DDAH1 reversely regulated β‐catenin expression in GC, it is tempting to speculate that one route by which loss of DDAH1 promotes EMT may be through the activation of β‐catenin. To explore this, we transiently knocked down β‐catenin in the AGS‐pshDDAH1 cells and looked for expression pattern changes in those EMT‐associated markers. Figure 5E shows that treatment of AGS‐pshDDAH1 with β‐catenin siRNA effectively restored the expression of epithelial markers (E‐cadherin and ZO‐1), but markedly reduced the expression of both mesenchymal markers (vimentin and N‐cadherin) and EMT repressor Snail as compared with the scrambled siRNA‐transfected control cells (Lane 4 versus Lane 3). Such acquisition of epithelial characteristics also translated into less aggressive phenotypes as reflected by decreased cell migration and invasion (Fig. 5F). These data suggest that the effect of DDAH1 knockdown on EMT induction is mediated primarily by the enhanced β‐catenin.

### 3.5 DDAH1 regulates β‐catenin expression *via* WNT/GSK‐3β pathway

**Figure 6:**
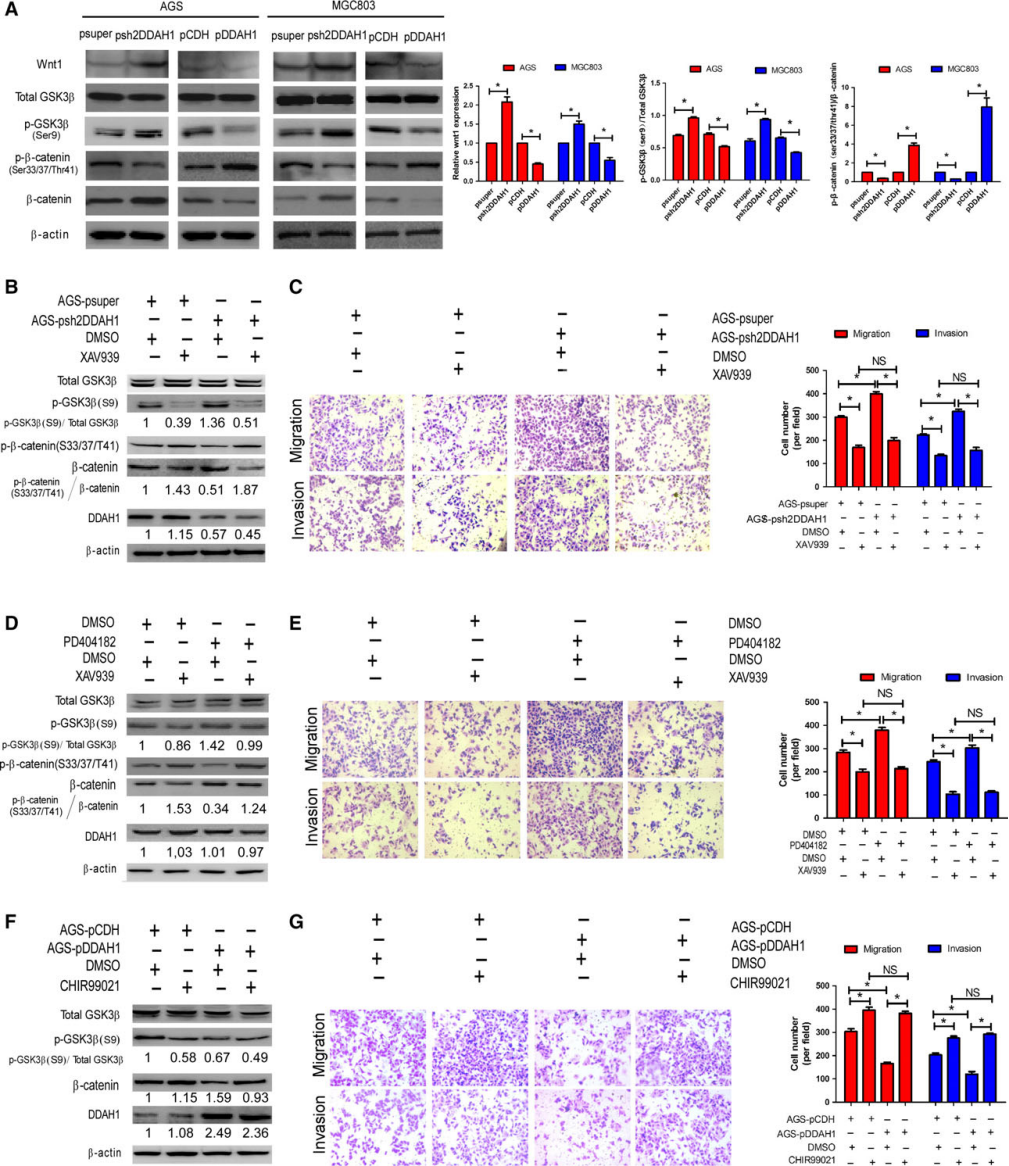
DDAH1 regulated β‐catenin expression *via* Wnt/GSK‐3β pathway. (A) Representative western blot analysis showing that Wnt1, p‐GSK‐3β (Ser9), and β‐catenin inversely correlated with DDAH1 expression, whereas p‐β‐catenin (Ser33/37/Thr41) positively correlated with DDAH1 expression. The histograms indicate the levels of the Wnt1 protein determined from three independent experiments after normalization to β‐actin, and the levels of phosphorylated GSK‐3β and β‐catenin were normalized to their relative total proteins (*P < 0.05). (B) The stimulatory effect of DDAH1 knockdown on β‐catenin expression was inhibited by XAV939 (a Wnt inhibitor). (C) The stimulatory effect of DDAH1 knockdown on cell migration and invasion was diminished by XAV939. The representative images were shown, and the data were expressed as mean ± SD (*P < 0.05; NS, no statistical significance). (D) The stimulatory effect of pharmacological inhibition of DDAH1 by PD404182 on β‐catenin expression was suppressed by XAV939 (a Wnt inhibitor). (E) The stimulatory effect of pharmacological inhibition of DDAH1 on cell migration and invasion was diminished by XAV939. The representative images were shown, and the data were expressed as mean ± SD (*P < 0.05; NS, no statistical significance). (F) The inhibitory effect of DDAH1 overexpression on β‐catenin expression was reversed by CHIR99021 (a Wnt agonist). (G) The inhibitory effect of DDAH1 overexpression on cell migration and invasion was reversed by CHIR99021. The representative images were shown, and the data were expressed as mean ± SD (*P < 0.05; NS, no statistical significance).

Glycogen synthase kinase‐3β (GSK‐3β)‐dependent control of β‐catenin stability or degradation is thought to be the central regulatory mechanism of the canonical Wnt signaling pathway (Clevers, 2006). In the presence of Wnt, GSK‐3β is phosphorylated and inhibited, thus preventing targeting of β‐catenin for degradation. More intriguingly, Wnt signal is found to be able to inhibit GSK‐3β‐mediated phosphorylation and consequently enhances Snail protein level leading to EMT in certain cell types (Yook *et al*., 2005, 2006; Zhou *et al*., 2004). Therefore, we were interested to determine whether Wnt/GSK‐3β pathway is involved in the regulation of β‐catenin induced by the loss of DDAH1. We found that knockdown of DDAH1 in the GC cells increased the levels of Wnt1, enhanced phosphorylation of GSK‐3β at Ser9, and reduced the phosphorylated forms of β‐catenin (Ser33/37/Thr41). Overexpression of DDAH1 displayed the opposite results (Fig. 6A). An important contribution of Wnt/GSK‐3β signaling to regulate β‐catenin expression was further supported by the observation that the addition of XAV939 (a WNT inhibitor) into the DDAH1‐knockdown AGS‐pshDDAH1 cells decreased GSK‐3β phosphorylation at Ser9, thus increasing the phosphorylated form of β‐catenin for degradation to reduce the total β‐catenin level (Fig. 6B), which was in accordance with the diminished ability of cells to migrate and invade (Fig. 6C). The aforementioned changes in the signaling molecules and cell migratory and invasive abilities were further confirmed when the AGS cells were treated with DDAH1‐specific inhibitor PD404182 rather than shRNAi‐knockdown cells (Fig. 6D,E). Conversely, treatment of the DDAH1‐overexpressing AGS‐pDDAH1 cells with CHIR99021 (a WNT agonist or GSK‐3β inhibitor) inactivated GSK‐3β by binding to it and subsequently disabled its phosphorylation, which led to diminished β‐catenin phosphorylation, therefore restoring the expression of total β‐catenin initially inhibited by DDAH1 overexpression (Fig, 6F). In line with this, the migratory and invasive capabilities of the AGS‐pDDAH1 cells in the presence of CHIR99021 substantially increased (Fig, 6G). Collectively, these results suggest that the effect of DDAH1 on β‐catenin stabilization or degradation is mediated, at least in part, through Wnt/GSK‐3β signaling.

## 4 Discussion

This study demonstrates that DDAH1 is frequently downregulated in clinical GC samples and its low expression is closely associated with more lymph node metastasis, lower histological differentiation, and poorer clinical outcome. In the experimental systems, forced expression of DDAH1 in the GC cells suppressed cell migration and invasion *in vitro* as well as metastatic potential *in vivo*. DDAH1 overexpression inhibited EMT process by increasing β‐catenin degradation through the attenuation of Wnt/GSK‐3β signaling. In contrast, knockdown of DDAH1 produces the opposite effects. These findings clearly implicate that DDAH1 functions as a tumor suppressor in GC and may be exploited as a prognostic biomarker in GC.

We found that overexpression or knockdown of DDAH1 in AGS or MGC803 cells did not cause any changes in the rate of proliferation *in vitro* nor the growth rate *in vivo*. Kostourou *et al*. (2002) showed that DDAH1 did not influence glioma cell proliferation *in vitro*. However, there are a few reports demonstrating that DDAH1 could promote glioma growth by angiogenesis *in vivo* (Boult *et al*., 2011; Kostourou *et al*., 2002; Wojciak‐Stothard *et al*., 2007). The discrepancy may be attributed to the different cell types.

EMT is associated with dissemination of cancer, thereby facilitating tumor progression (Brabletz, 2012; Iwatsuki *et al*., 2010). During EMT, cancer cells decrease cell–cell junctions and gain the mesenchymal characteristics, thus enhancing their abilities of migration and invasiveness (Thiery, 2002; Thiery *et al*., 2009). In our work, we found that DDAH1 expression was positively correlated with the epithelial markers (E‐cadherin and ZO‐1) and reversely with the mesenchymal markers (vimentin and N‐cadherin). In agreement with this result, DDAH1 has been reported to increase expression of E‐cadherin as well as to decrease the levels of a‐SMA, collagen I, and fibronectin in HK‐2 cell (Liu *et al*., 2016), indicating that DDAH1 may involve in reversal of EMT progression. Furthermore, we also found that DDAH1 decreased the level of transcriptional factor Snail, known to repress E‐cadherin expression by binding to the E‐box regulatory element within the E‐cadherin gene (Thiery, 2002; Thiery *et al*., 2009). Taken together, we assume that DDAH1 reduces GC cell migration and invasion *in vitro* and metastatic potential *in vivo* likely through the inhibition of an EMT process.

While the permissive signal that regulates the EMT program in GC remains largely unknown, DDAH1 has been reported to be involved in mediating Wnt/β‐catenin pathway (Liu *et al*., 2016). Aberrant Wnt/β‐catenin signaling is known to play an important role in the initiation and maintenance of GC (Hidaka *et al*., 2013; Ju *et al*., 2014). While Wnt signaling is activated, β‐catenin is stabilized and accumulates in the cytoplasm and then migrates to the nucleus where it binds to the T‐cell factor/lymphoid enhancer‐binding factor (TCF/LEF) to promote transcription of various target genes, inducing those related to EMT (Clevers, 2006; Clevers and Nusse, 2012; Huang *et al*., 2014). In the present study, we found that DDAH1 overexpression significantly decreased the level of β‐catenin, whereas its knockdown produced the opposite effect. This DDAH1‐dependent β‐catenin level change was accompanied by the alteration of GC cell aggressive phenotypes. Such important link is further supported by our finding that siRNA knockdown of β‐catenin in AGS‐pshDDAH1 cells reversed the EMT and reduced the cell migratory and invasive capabilities.

We next aimed to identify the signaling mechanism by which DDAH1 mediates β‐catenin in GC cells. The cellular localization and accumulation of β‐catenin are known to be tightly regulated by Wnt proteins. In the absence of Wnt, β‐catenin is phosphorylated by GSK‐3β, resulting in its phosphorylation, ubiquitination, and proteasomal degradation (Buescher and Phiel, 2010; Chigita *et al*., 2012; Takahashi‐Yanaga, 2013). In the presence of Wnt, β‐catenin is activated through the canonical Wnt pathway and ultimately leads to GC progression (Clevers and Nusse, 2012). Our results showed that DDAH1 expression was reversely associated with the levels of Wnt1 and p‐GSK‐3β (Ser9) but positively related to the level of p‐β‐catenin (Ser33/37/Thr41). We also found that treatment with a specific Wnt inhibitor (XAV939) suppressed the stimulatory effect of DDAH1 knockdown on β‐catenin level, migration, and invasion in the GC cells. In addition, a GSK‐3β‐specific inhibitor CHIR99021 can reverse the DDAH1‐induced suppression on intracellular β‐catenin level, cell motility, and invasiveness. Together, the present results point toward a concept that DDAH1 mediates β‐catenin *via* Wnt/GSK‐3β signaling pathway in GC.

In summary, this is the unprecedented study exploring the role of DDAH1 in GC and demonstrates for the first time that DDAH1 may act as a tumor suppressor specifically in GC. Downregulation of DDAH1 in GC, which is strongly correlated with tumor progression and clinical prognosis, merits further development as a diagnostic and prognostic biomarker. Moreover, while a strategy to enhance DDAH1 functionality is difficult to accomplish, any approaches to increasing the level or activity for tumor suppression may possess a therapeutic value.

## Author contributions

JY, JX, WC, and XL involved in conception and design. JY, JX, YL, QH, and JH involved in development of methodology. JY, JX, YL, QH, JH, JW, and WZ involved in acquisition of data (acquired and managed patients, provided facilities, etc.). JY, JX, XL, WC, and XL analyzed and interpreted the data (e.g., statistical analysis, biostatistics, computational analysis). JY, JX, XL, WC, and XL involved in writing, review, and/or revision of the manuscript. JY, JX, YL, and QH provided administrative, technical, or material support (i.e., reporting or organizing data, constructing databases). WC and XL supervised the study.

## Supporting information


**Table S1.** Oligonucleotides used for cloning and qRT‐PCR.
