# Supplementary material for: DDAH1 mediates gastric cancer cell invasion and metastasis via Wnt/β‐catenin signaling pathway
Source: Mol Oncol. 2017 Jun 22;11(9):1208–24. doi: 10.1002/1878-0261.12089 (PMC5579338; doi:10.1002/1878-0261.12089)
Supplement: Supplementary file 1 — Table S1. Oligonucleotides used for cloning and qRT‐PCR. [file MOL2-11-1208-s001.docx]

Supplementary Table S1. Oligonucleotides used for cloning and qRT-PCR

| **Oligonucleotides** | **Sequences (5′-3′)** |
| --- | --- |
| **shDDAH1** | |
| Sh1DDAH1- F | GATCCCCCATGTCTGAACTGGAAAAGGTTTCAAGAGAACCTTTTCCAGTTCAGACATGTTTTTA |
| Sh1DDAH1-R | AGCTTAAAAACATGTCTGAACTGGAAAAGGTTCTCTTGAAACCTTTTCCAGTTCAGACATGGGG |
| Sh2DDAH1- F | GATCCCCCAGCTACGACAAACTCACTGTTCAAGAGACAGTGAGTTTGTCGTAGCCATGTTTTTA |
| Sh2DDAH1- R | AGCTTAAAAACAGCTACGACAAACTCACTGTCTCTTGA ACAGTGAGTTTGTCGTAGCCATGGGG |
| **DDAH1 cloning** |  |
| DDAH1-F | CGGAATTCGCCACCATGGCCGGGCTCGGCCACC |
| DDAH1-R | CGGGATCCTCACTTGTCGTCATCGTCTTTGTAGTCGGAGTCTACTTTCTTGTTA |
| **Real-time RT-PCR** | |
| β-actin-F | CCTGGCACCCAGCACAAT |
| β-actin-R | GGGCCGGACTCGTCATACT |
| DDAH1-F | ACTCACTGTGCCTGATGACA |
| DDAH1 -R | TCCAGTTCAGACATGCTCA |
| E-cadherin-F | GTCTGTCATGGAAGGTGCT |
| E-cadherin-R | TACGACGTTAGCCTCGTTC |
| ZO-1- F | AGATTTGGAACTCCCTGAGA |
| ZO-1- R | GCTTCTGTTACTAGGATCCA |
| Vimentin-F | CCACGAAGAGAAATCCAGG |
| Vimentin-R | CAGAGAGGTCAGCAAACTTGG |
| N-cadherin-F | ATCACAGTGACAGATGTCA |
| N-cadherin-R | AACGCAGTGTACAGAATCAG |
| Snail-F | CCTTCTCTAGGCCCTGGCT |
| Snail-R | AGGTTGGAGCGGGTCAGC |
| β-catenin--F | GACAGAGTTACTTCACTCTA |
| β-catenin--R | CATTGGCTCTGTTCTGAAGA |
| **β-catenin siRNA** | |
| sense | UUGUACCGGAGCCCUUCACTT |
| Antisense | GUGAAGGGCUCCGGUACAATT |
| **Nontargeting control siRNA** | |
| sense | UUCUUCGAACGUGUCACGUTT |
| Antisense | ACGUGACACGUUCGGAGAATT |
